# OP40 Publication Priorities In Pharmaceutical Journals Of Epidemics Proposed For Elimination By The Sustainable Development Goals

**DOI:** 10.1017/S0266462325100986

**Published:** 2025-12-29

**Authors:** Julia Soto Rizzato, Marcus T. Silva, Tais Galvao

## Abstract

**Introduction:**

Scientific journals are essential for scientific dissemination, indirectly reflecting research interests and funding. Epidemics targeted to end by the Sustainable Development Goals (SDG) 3.3 (AIDS, malaria, tuberculosis) still have significant impacts, requiring innovative health technologies. Monitoring publications on these epidemics can guide strategies to prioritize research and funding. We investigated publication priorities about the SDG 3.3 in pharmaceutical journals and associated factors.

**Methods:**

This cross-sectional study included journals in the pharmacology and pharmacy category of the Journal Citation Reports (JCR) in 2022. The primary outcome was publication priority on AIDS, malaria, and tuberculosis, defined as greater than or equal to three percent. Bibliometric indicators were obtained from the JCR, and the number of publications in each journal up to 2022 were collected using the Web of Science Core Collection via EndNote. The editors-in-chief countries were identified on journal websites and classified as low- and middle-income or not according to the World Bank. Prevalence ratios (PR) and 95 percent confidence intervals (CI) of the outcome and journal factors were calculated by Poisson regression.

**Results:**

Of 362 eligible journals, eight were excluded due to discontinuation or missing editor information. Most journals started between 1991 and 2010 (39.4%) and were open access (50.7%). Sixty-one editors-in-chief were from low- and middle-income countries (17.3%), with most of their journals belonging to the JCR’s Emerging Sources Citation Index (39.0%; p<0.001) and being open access (22.9%; p=0.005). More recent journals had more editors-in-chief from developing countries (34.5%; p<0.001) and higher publication priority on the SDG 3.3 (PR=2.32; 95% CI: 1.47, 3.66). Journals with such editors published more on the SDG 3.3 (PR=1.57, 95% CI: 1.10, 2.23) and had lower impact factor (2.33 [standard deviation 2.63] versus 4.10 [standard deviation] 7.39; p=0.066).

**Conclusions:**

Journals that were founded more recently and had editors from developing countries published more articles on the SDG 3.3. Increasing the diversity of editorial boards in scientific journals could be an important strategy to promote the dissemination of health technologies aimed at addressing these neglected diseases and achieving the SDG by 2030.

